# Biochemical and molecular characterisation of exogenous cytokinin application on grain filling in rice

**DOI:** 10.1186/s12870-018-1279-4

**Published:** 2018-05-21

**Authors:** Binay Bhushan Panda, Sudhanshu Sekhar, Sushant Kumar Dash, Lamboder Behera, Birendra Prasad Shaw

**Affiliations:** 1 Institute of Life Sciences, Bhubaneswar, Odisha India; 2 National Rice Research Institute, Cuttack, Odisha India

**Keywords:** Cytokinin oxidase, Cytokinin treatment, 6-Benzylaminopurine, Cell cycle regulators, Chromosomal endoreduplication, *Oryza sativa*

## Abstract

**Background:**

Poor filling of grains in the basal spikelets of large size panicles bearing numerous spikelets has been a major limitation in attempts to increase the rice production to feed the world’s increasing population. Considering that biotechnological intervention could play important role in overcoming this limitation, the role of cytokinin in grain filling was investigated based on the information on cell proliferating potential of the hormone and reports of its high accumulation in immature seeds.

**Results:**

A comparative study considering two rice varieties differing in panicle compactness, lax-panicle Upahar and compact-panicle OR-1918, revealed significant difference in grain filling, cytokinin oxidase (CKX) activity and expression, and expression of cell cycle regulators and cytokinin signaling components between the basal and apical spikelets of OR-1918, but not of Upahar. Exogenous application of cytokinin (6-Benzylaminopurine, BAP) to OR-1918 improved grain filling significantly, and this was accompanied by a significant decrease in expression and activity of *CKX*, particularly in the basal spikelets where the activity of CKX was significantly higher than that in the apical spikelets. Cytokinin application also resulted in significant increase in expression of cell cycle regulators like cyclin dependent kinases and cyclins in the basal spikelets that might be facilitating cell division in the endosperm cells by promoting G1/S phase and G2/M phase transition leading to improvement in grain filling. Expression studies of type-A response regulator (*RR*) component of cytokinin signaling indicated possible role of *OsRR3*, *OsRR4* and *OsRR6* as repressors of *CKX* expression, much needed for an increased accumulation of CK in cells. Furthermore, the observed effect of BAP might not be solely because of it, but also because of induced synthesis of trans-zeatin (tZ) and N^6^-(Δ^2^-isopentenyl)adenine (iP), as reflected from accumulation of tZR (tZ riboside) and iPR (iP riboside), and significantly enhanced expression of an isopentenyl transferase (*IPT*) isoform.

**Conclusion:**

The results suggested that seed-specific overexpression of *OsRR4* and *OsRR6*, and more importantly of *IPT9* could be an effective biotechnological intervention towards improving the CK level of the developing caryopses leading to enhanced grain filling in rice cultivars bearing large panicles with numerous spikelets, and thereby increasing their yield potential.

**Electronic supplementary material:**

The online version of this article (10.1186/s12870-018-1279-4) contains supplementary material, which is available to authorized users.

## Background

Rice is a staple crop for the people of Asian and African regions, which are most populated as well compared with other regions of the world [1]. Demand for cereal brought in green revolution in rice in the Asian countries in the last half of the twentieth century, mid 1960s to mid 1990s, doubling its worldwide production from 6 t/ha to 10 t/ha [2, 3]. However, the rate of 2.7% increase in annual yield achieved in 1980s has got drastically reduced to 1.1% evaluated until 1990s [3]. This is in sharp contrast to the fact that the world population is going to increase to 9.6 billion by 2050 [4] from the current figure of 7.3 billion [5]. The rapid increase in world population coupled with economic development is putting increasing demand to increase food production [6], and according to one estimate, 56% more rice is required to be produced in next three decades to meet the demand [7].

The first green revolution was primarily the result of genetic improvement of the rice varieties, in addition to expansion of the land area under irrigation and judicious use of fertilizers and agro-chemicals [3, 8]. The major genetic improvement was in terms of increase in yield potential because of the release of short-stature semi-dwarf genotypes of both the rice sub-species, Japonica and Indica, which increased the harvest index from 0.3 to 0.5 [3, 9]. To increase the yield further, keeping in view the growing population of the world and narrowing down of the gap between yield potential and farmers’ yield [10], the new plant type (NPT) was designed to increase the harvest index approximately to 0.6 by modifying the architecture of the plant, including the number of spikelets on the panicle [9]. The effort to increase the number of spikelets on panicles was made to enhance the sink capacity with the aim of breaking the yield ceiling. Breeding effort in this regard resulted in cultivars with large panicles bearing numerous spikelets, including the NPT of the International Rice Research Institute (IRRI) [10] and ‘super hybrid rice’ in China [11, 12]. The yield potential of these cultivars is, however, not harnessed due to poor filling and maturation of grains in the spikelets, particularly of those occupying inferior (basal) position on the panicle [10, 13, 14]. Poor filling of grains is also seen in the spikelets of other large panicle rice varieties/cultivars [15, 16].

Apart from seed setting, the grain yield in rice to a great extent is thus determined by the numbers of spikelets borne on the panicles, which in itself is a quantitative trait [17]. So far 17 QTLs have been identified that determines the number of spikelets on rice panicles [17]. Panicle development is a transition from vegetative to reproductive phase wherein the shoot apical meristem is transformed into panicle meristem that differentiates into primary, secondary, or even tertiary lateral meristems producing primary, secondary and tertiary branches, respectively, with each lateral meristem terminating into a spikelet/floret [17, 18]. The factors determining the size of panicle or inflorescence and the number of spikelets on it are largely unknown. However, one of the most effective QTLs identified and cloned for grain number is Gn1a encoding cytokinin oxidase/dehydrogenase (OsCKX2) that degrades cytokinins [19]. The loss of function mutation of Gn1a (*OsCKX2*) was also demonstrated to be the reason for a greater number of spikelets in the cultivar compared with that having functional *OsCKX2* [19]. *Gn1a* has also been recently demonstrated as a negative regulator of grain number by mutating the gene using CRISPR/Cas9 gene editing technique, which resulted in significant increase in number of flowers (spikelets) per panicle in the mutant lines of Zhonghua 11 Japonica caultivar [20]. In addition, mutations of the genes like *DEP1* (*DENSE AND ERECT PANICLE1*) and *IPA1* (*IDEAL PLANT ARCHITECTURE1*) that control panicle architecture and tillering, respectively, by CRISPR/Cas9 editing also led to increase in the number of spikelets per panicle, suggesting pleotropic effects of the yield related genes [20].

Considering the fact that auxin inhibits axillary bud growth, while cytokinin relieves the growth inhibition [17, 21], a greater number of spikelets in the cultivar having non-functional *OsCKX2* compared with that having functional *OsCKX2* [19] is plausible. In addition to promoting axillary buds growth, the plant hormone cytokinin is known for its regulation of many cellular and developmental processes, such as induction of cell division and de novo shoot formation [22], flowers and fruit development, and seed germination [23], shoot and root branching [24], photomorphogenic development and vascular differentiation [25, 26], and chloroplast biogenesis and leaf senescence [27]. In these contexts, the role of OsCKX becomes very important because it could regulate the level of the hormone cytokinins in cells and tissues, as is evident from decrease/increase in cytokinin contents in *CKX* overexpressing/knockdown plants [28, 29], in addition to regulation of the cellular levels of the hormone by biosynthetic processes and conversion to functionally inactive forms through N- and O-glycosylation [30, 31].

In addition to panicle development, important role of cytokinin and other plant hormones has also been indicated in development of a spikelet to a mature grain after fertilization. The process of grain development includes increase in endosperm cell numbers and filling of the endosperm cells with starch. Both these processes are slow in the basal than that in the apical spikelets of large panicle rice, resulting in a poor filling of grains in the former. However, spikelet-thinning treatment by removing some of the apical spikelets, which increased filling of grains in the basal spikelets, has proved it sufficiently that each spikelet is competent to get filled well post-fertilization producing well-developed grain [32, 33]. This proves beyond doubt that it is not the supply of carbohydrates or the source, but the sink strength, the product of sink size and sink activity, that limits the grain filling process and ultimately grain development by putting physiological constrain on the import of assimilates [34]. However, the intrinsic factor(s) causing differential grain filling in rice spikelets based on their spatial location is by and large still illusive. Reports of inhibition of grain filling by ethylene and its promotion by abscisic acid (ABA) and auxin in the basal spikelets [35, 36], nevertheless, indicate a possible role of plant hormones in differential grain filling in the apical and basal spikelets. Furthermore, Yang et al. [37] have indicated that both ABA and ethylene are required for post-fertilization growth of ovule into grain, but a high ABA to ethylene ratio in the spikelets leads to a faster rate of grain filling. There are also reports of involvement of gibberellins and ABA in regulation of grain development [38, 39]. Keeping in view the role of cytokinin in promoting cell division, and the finding that generally a high level of cytokinin is maintained in the endosperm during seed development, it has been suggested that action of the hormone might be essential to promote endosperm cell division that is required during the initial period of the seed development process [40, 41]. Studies have also found that the cytokinin level in spikelets significantly increases with seed development in wheat, rice and maize [35, 42–45].

Despite a clear indication of regulatory role of plant hormones, particularly of cytokinin, in seed development, a clear understanding on mechanistic details of the hormone action in the process is not yet fully delineated. In the present study the objectives were to understand the involvement and role of cytokinin in grain filling by conducting a comparative study of activities and expression of cytokinin oxidase (CKX), expression of cytokinin signaling components and other grain filling related parameters in the apical and basal spikelets of rice varieties/cultivars that differed in grain filling in the spikelets borne on the basal region of panicle, and to see the influence of external application of cytokinin on the grain filling process in the cultivar showing differential grain filling in the apical and basal spikelets.

## Methods

### Rice cultivars and cytokinin application

Based on our previous experience of grain filling in various Indica rice (*Oryza sativa* L.) cultivars [15, 16], two of them, Upahar and OR-1918 that showed good and poor grain filling, respectively were selected for the present study. Seeds of these plants were collected from OUAT (Orissa University of Agriculture and Technology), Bhubaneswar, India. The seeds were germinated and grown on nursery bed, and almost 30 days old seedlings were transplanted during Kharif season of 2015. For the comparative study of yield parameters and enzyme activity, the seedlings of the two cultivars were transplanted in the field facility at National Rice Research Institute (NRRI). Plantation was done in 3 × 3 m split design plots with three replicates. The plantation was done at 20 × 15 cm spacing. Chemical fertilizers in the form of urea (N) muriate of potash (K) and single superphosphate (P) were applied at the rate 80(N):40(K):40(P) kg ha^− 1^ in split doses; 50% of N and K, and all of P during transplantation, 25% of N during tillering and the remaining 25% of N and half of K during panicle initiation stage. The water level in the field was maintained ~ 6 cm throughout before maturity.

For the study of effect of cytokinin on grain filling, panicle morphology and various biochemical parameters, only the rice cultivar OR-1918 was considered. The seedlings grown as above were transplanted into experimental pots in the Rabi season (2016). The pots were circular made of 1.25″ thick RCC structure measuring 42″ in diameter and 23″ in depth. Ten pots were placed in two lanes in open place in the Institute’s campus avoiding any shadow. These were filled with a mixture of sandy loam soil and farmyard manure (3:1) leaving ~ 6″ space from top and watered till the soil got inundated. The volume of water required to fill the individual pots completely, leaving 3″ space from top, was recorded. Water in the individual pots was allowed to evaporate until the soil was just wet. At this point each pot was filled with ~ 2″ of water above the soil and 9 seedlings were transplanted at the rate one seedling per square foot. The pots were irrigated with water regularly maintaining the water level of the individual pots ~ 3″ from top all the time. Commercially available fertilizers containing N, K and P were applied as above. Synthetic cytokinin BAP (6-Benzylaminopurine) was applied exogenousely for uptake by root. The hormone was applied to one of the rows of the pots to a final concentration of 50 μM at the late booting stage, i.e. just before emergence of the inflorescence, taking into consideration the total volume of water in each pot. BAP treatment concentration of 50 μM was considered based on earlier report of use of such concetration of the hormone for exogenous application [46].

### Spikelet sampling and yield parameters study

Sampling of the spikelets for various analyses was based on their anthesis period. In rice, anthesis starts from apical region of the panicle and progress towards the basal region, getting completed in about 6 days. The spikelets occupying the primary branches at the apical region reach anthesis first. It takes approximately 3 days for the spikelets on the middle of the panicle to reach anthesis. The day on which the anthesis begins either on the apical or basal part of the panicle was referred ‘0’ day after anthesis (DAA). The main shoot and the primary tillers were marked with colour threads on the day on which they showed emergence of panicle and the spikelets on the top primary branches in the apical region showed anthesis. The spikelets from the apical and basal regions of the panicle were excised on their respective 3, 6, 9 and 12 days post anthesis, and these were referred as 3, 6, 9 and 12 DAA samples, respectively. The samples collected in replicates in 15 mL falcon tubes were dipped in liquid nitrogen immediately and stored at − 80 °C until use. The main tillers and the primary branches not used for sampling of spikelets were allowed to progress to maturity for the study of yield parameters, which included panicle length, panicle weight, total grain numbers, percentage of filled grain, 20 apical grain weight, 20 basal grain weight and yield per plant.

### Cytokinin oxidase (CKX) activity

Cytokinin oxidase activity was studied in the spikelets of Upahar and OR-1918 sampled on 3, 6, 9 and 12 DAA from the apical and basal regions of the panicle. The activity of the enzyme was estimated following Frébort et al. [47]. The frozen spikelet samples collected as above were crushed in liquid nitrogen separately using porcelain mortar and pestle. Three biological replicates were taken. The powdered tissue of the individual samples was suspended in the ezyme extraction solution made of 0.1 M Tris-HCl buffer (pH 8.0), 1 mM phenylmethylsulfonyl fluoride, 1% protease inhibitor (Focus Protease Arrest™, Cat. #786108F, G-Biosciences), 2 mM dithiothreitool and 1% Triton X-100 in 3:1 (*v*/*w*) ratio in 2 mL microfuge tube and kept over ice for 30 min with gentle shaking several times. The tissue suspension was centrifuged for 10 min at 12000×g to pellet out the cell debris. The supernatant was used for CKX activity assays. The enzymatic reaction for each biological sample extract was carried out in triplicate. Each enzymatic reaction mixture contained 100 μL enzyme extract, 0.5 mM DCPIP (2,6-dichlorophenol indophenol, an electron acceptor) and 0.15 mM iP in a final volume of 0.6 mL 75 mM Tris-HCl buffer (pH 8.0). The substrate iP was added last and the mixture was incubated for 1 h at 37 °C for the enzymatic reaction to proceed. The progress of the reaction in the individual tubes was stopped by adding 0.3 mL of 40% (*w*/*v*) TCA (trichloroacetic acid). After 15 min the reaction mixture was centrifuged for 5 min at 12000×g to remove the precipitate. The supernatant was collected and to it was added 0.2 mL of 4-aminophenol (2% w/v solution in 6% TCA). After 5 min the reation solution was transferred in a quartz cuvette for measuring the absorbance at 352 nm. The enzymatic degradation product 3-methyl-2-butenal reacts with 4-aminophenol under acidic conditions to form Schiff base ((4-hydroxyphenylimino)-3-methyl-2-buten) having a molar absorption co-efficient of ε_352_ = 15.2 mM^− 1^ cm^− 1^. The enzyme activity was calculated considering the protein concentration of the enzyme extract and the Schiff base produced. Protein concentration in the individual enzyme extracts was determined using Bradford reagent [48].

### Gene expression studies

Expression studies of all the genes under consideration were done by RT-PCR and RT-qPCR. Extraction of total RNA from the individual spikelet samples (stored at − 80 °C) was done using TRIZOL (Thermo Fisher Scientific) as per the instruction manual. Pelleted RNA of the individual samples was dissolved in DEPC-water separately and the concentration of RNA in each was measured using a nano-drop spectrophotometer. The RNA extracted from each sample was stored at − 80 °C until used for the expression studies. QuantiTect Reverse Transcription Kit from Qiagen was used to reverse transcribe the RNA to cDNA following the kit’s instruction manual. The individual cDNA preparations were used for gene expression studies. The cDNA sequence of the individual genes was extracted from the publically accessible websites of Rice Genome Project Annotation (http://rice.plantbiology.msu.edu/index.shtml), NCBI (http://www.ncbi.nlm.nih.gov), and EnsemblePlants (http://plants.ensembl.org/index.html). The primers for the expression study of the individual genes were designed using primer-BLAST software (http://www.ncbi.nlm.nih.gov/tools/primer-blast/) at the NCBI site (Additional file 1) considering the sequences retrieved from all the three sites. DNA engine (Bio-Rad) thermocycler was used for PCR. The reagents used were PCR nucleotide mix (Promega, C1141) and GoTaq® DNA Polymerase (Promega, M8291). The programme set for each PCR was 94 °C for 3 min, 30 cycles each of 94 °C for 1 min, 59 °C for 1 min and 72 °C for 1 min, and finally 72 °C for 10 min and then 4 °C were maintained. The PCR products were run on 2% agarose gel impregnated with ethidium bromide (0.2 μg mL^− 1^ agarose solution) and visualized under UV illumination on a trans-illuminator. *Actin* served as the reference gene. Based on the results of RT-PCR, RT-qPCR was performed for the desired genes on LightCycler® 480 Real-Time PCR System (Roche) using Brilliant III Ultra-Fast SYBR® Green QPCR Master Mix (Agilent Technologies). The relative level of cDNA templates of the concerned gene was obtained following Pfaffl [49] and considering *Actin* cDNA templates as the reference level. The results were expressed as relative level (2^-∆∆C^_T_) in Apical/Basal spikelets or BAP treated/control spikelets with value less than one representing a lower expression in apical compared with basal or that in treated compared with control.

### Endosperm cell counting and flowcytometeric analysis

The spikelets of 12 DAA apical and basal samples were kept submerged for 48 h in Carnoy’s solution (9:3:1 *v*/v/v of absolute alcohol, glacial acetic acid and chloroform), and subsequently transferred to 70% ethanol diluted with water till further processing [50]. Following this, the hulls were removed and the caryopses were sequentially soaked in 50% and 25% ethanol in water for 1 h each and finally soaked in Milli-Q water for 5–7 h. Subsequently, individual caryopses were placed in 1 mL solution of 0.1% *w*/*v* cellulase (C1794 Sigma Aldrich), pH 5.0, and incubated at 40 °C in an orbital shaker for 5–6 h at 250 rpm. The endosperm cells liberated were collected by passing the hydrolyzed materials through 150 μ nylon mesh [51]. The endosperm cells liberated (10 μL) from the individual caryopses were transferred in Delafield’s haematoxylin solution and kept for 30 h for staining. The stained cells were centrifuged out and then suspended in Milli-Q water and centrifuged several times at 150 x g to remove the excess stain. The stained cells were finally pelleted out and suspended in 100 μL Milli-Q water. An aliquot of each cell suspension was then diluted 1000 times and the cells were counted using RBC counting chamber. Flowcytometric analysis was done separately using the unstained cells. For this, 300 μL of 1000 times diluted endosperm cells preparation was taken and equilibrated with PBS buffer (pH 7.0) having 1% Triton X-100 and then centrifuged at 150 x g to pellet out the nuclei. The pelleted nuclei were suspended in 300 μL of PBS buffer and stained with DAPI (5 μg mL^− 1^). Following this, the cells were sorted on BD LSR-Fortessa flow cytometer for the density of nuclear staining [51]. For each endosperm cell preparations about 10,000 nuclei were analyzed, and endosperm cell preparations from at least three individual caryopses from control or treated plants, or apical or basal spikelets were considered for counting.

### Microscopic analysis of endosperm cells

The caryopses were dissected out from 12 DAA apical and basal spikelets of BAP-treated and control plants. These were separately soaked overnight in fixing solution made of 50 mM potassium phosphate buffer (pH 6.8), 3.7% paraformaldehyde and 0.2% picric acid. After washing the fixed endosperm with phosphate buffer, these were dehydrated in gradients of aqueous ethanol and finally dehydrated in absolute alcohol followed by soaking in xylene following standard protocol. The xylene soaked caryopses were then infiltrated with paraffin for sectioning using microtome. The microtome sections, 7 μm in thickness, were mounted on glass slides. Paraffin coating was removed from the sections by washing with xylene. Finally, the slides with sections were soaked in DAPI (4′,6-diamidino-2-phenylindole) solution (300 ng mL^− 1^) for staining. The stained sections were visualized under fluorescence stereo-microscope for observing the cells and nuclei.

### Immuno localisation of cytokinins

Considering 9 DAA as the period of active metabolism, the apical and basal spikelets collected during this time from the control and BAP-treated OR-1918 cultivar were fixed in Carnoy’s solution [50] and processed to get the caryopses as mentioned in the cell counting and flowcytometeric analysis methodology above. Microtome sectioning of these caryopses followed the procedure outlined in the microscopy of endosperms section above. The sections were de-paraffinated by soaking in xylene and then rehydrated by saoking in ethanol solution in water with decreasing percentage of ethanol (100, 95, 70, 30 and 0%) for three min each, and then incubated in blocking buffer (0.1X PBS buffer pH 7.4, 0.25% *w*/*v* BSA, 0.1% Triton X-100, 2% goat serum) at rool temperature with three changes at 10 min interval. The sections were then immunolabelled for isopentenyladenosine (iPR) and trans-zeatin riboside (tZR) polyclonal antibodies separately by incubating at 4 °C overnight, and then at room temperature for 1 h. The antibodies were raised in rabit against iPR/tZR conjugated with BSA (Agrisera AB, Sweden). The antibodies used were diluted (1:100) beforehand with blocking buffer. The immuno-reacted sections were washed three times with PBS at 10 min interval and then incubated with secondary antibody (Goat anti-rabbit IgG [H&L], DYLight 594 conjugated) at room temperature for 2 h. The seconday antibody was diluted 1:200 beforehand with the blocking buffer. After incubation with the secondary antibody the sections were washed with blocking buffer (3 X 10 min) and PBS (2 X 10 min) and the whole section was visualized under a fluorescence stereo-microscope.

## Results

**Fig. 1:**
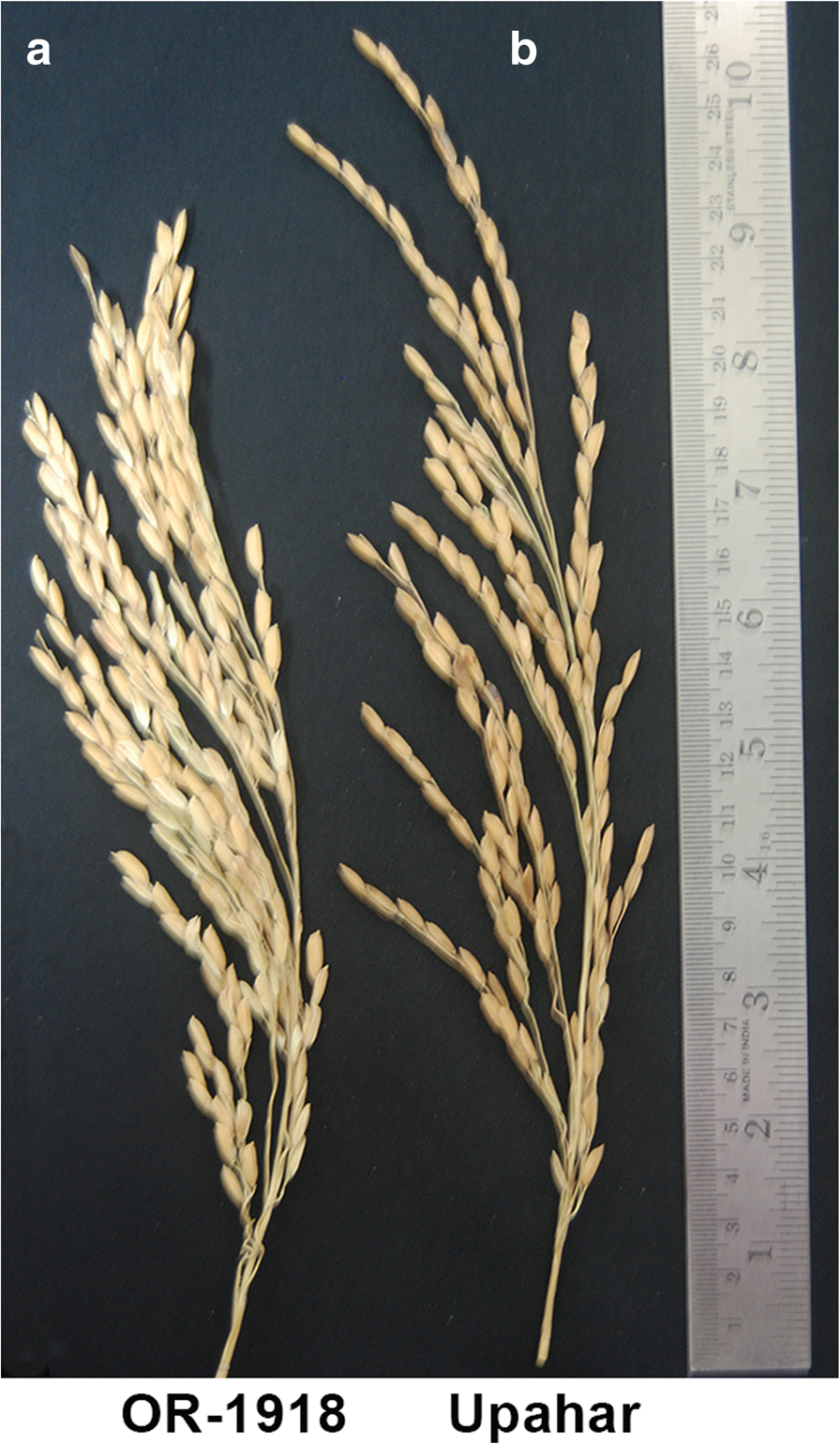
Representative pictures of compact (**a**) and lax (**b**) panicles collected from the plants grown in field

Significant differences in yield performance and panicle morphology were observed between the lax- and compact-panicle cultivars, Upahar and OR-1918, respectively (Table 1, Fig. 1). The spikelet numbers per panicle was much more in the compact-panicle OR-1918 than that in the lax-panicle Upahar (Table 1). In contrast, percentage of grain filling was much more in Upahar (91.60%) than in OR-1918 (56.23%). Exogenous application of cytokinin to OR-1918 significantly increased its grain filling percentage (Table 1). Besides, cytokinin treatment also increased the total number of grain on a panicle, the length of panicle, both apical and basal grain weight, and the grain yield per plant as a whole in OR-1918.

**Table 1:** Panicle morphology of OR-1918 and Upahar grown in field and of OR-1918 grown in experimental pots and exposed to 50 μM BAP

	Panicle wt. (gm)	Panicle length (cm)	Spikelets (number)	Filled gain (%)	20 apical grain wt. (mg)	20 basal grain wt. (mg)	yield/plant (gm)
Plants grown in field							
OR-1918	5.3 ± 0.2	25.5 ± 1.5	396 ± 13.45	56.23 ± 2.2	430.3 ± 8.6	244.3 ± 21.0	46.9 ± 2.48
Upahar	5.6 ± 0.76	27.06 ± 0.51	256 ± 10.0	91.60 ± 0.56	436.3 ± 5.5	412.6 ± 9.0	53.6 ± 1.97
‘t’ value at df = 4	0.73^ns^	0.47^ns^	9.2***	26.9***	1.01^ns^	12.7***	3.6*
OR-1918 grown in pots							
Control	5.38 ± 0.27	24.81 ± 0.98	403 ± 22	59.1 ± 2.8	423 ± 14	364 ± 21	47.9 ± 4.6
BAP	6.14 ± 0.19	26.43 ± 0.92	479 ± 25	72.2 ± 2.3	454 ± 16	392 ± 13	69.3 ± 6.2
‘t’ value at df = 18	7.3^***^	3.82^**^	27^***^	5.4^***^	4.6^***^	3.5^**^	6.19^***^

Data are mean ± SD. The ‘t’ values marked with ‘*’, ‘**’, or ‘***’ indicate that the difference between concerned two mean values is significant at *p* ≤ 0.05, *p* ≤ 0.01 or *p* ≤ 0.001 ***, respectively, while ‘ns’ indicates no significant difference

**Fig. 2:**
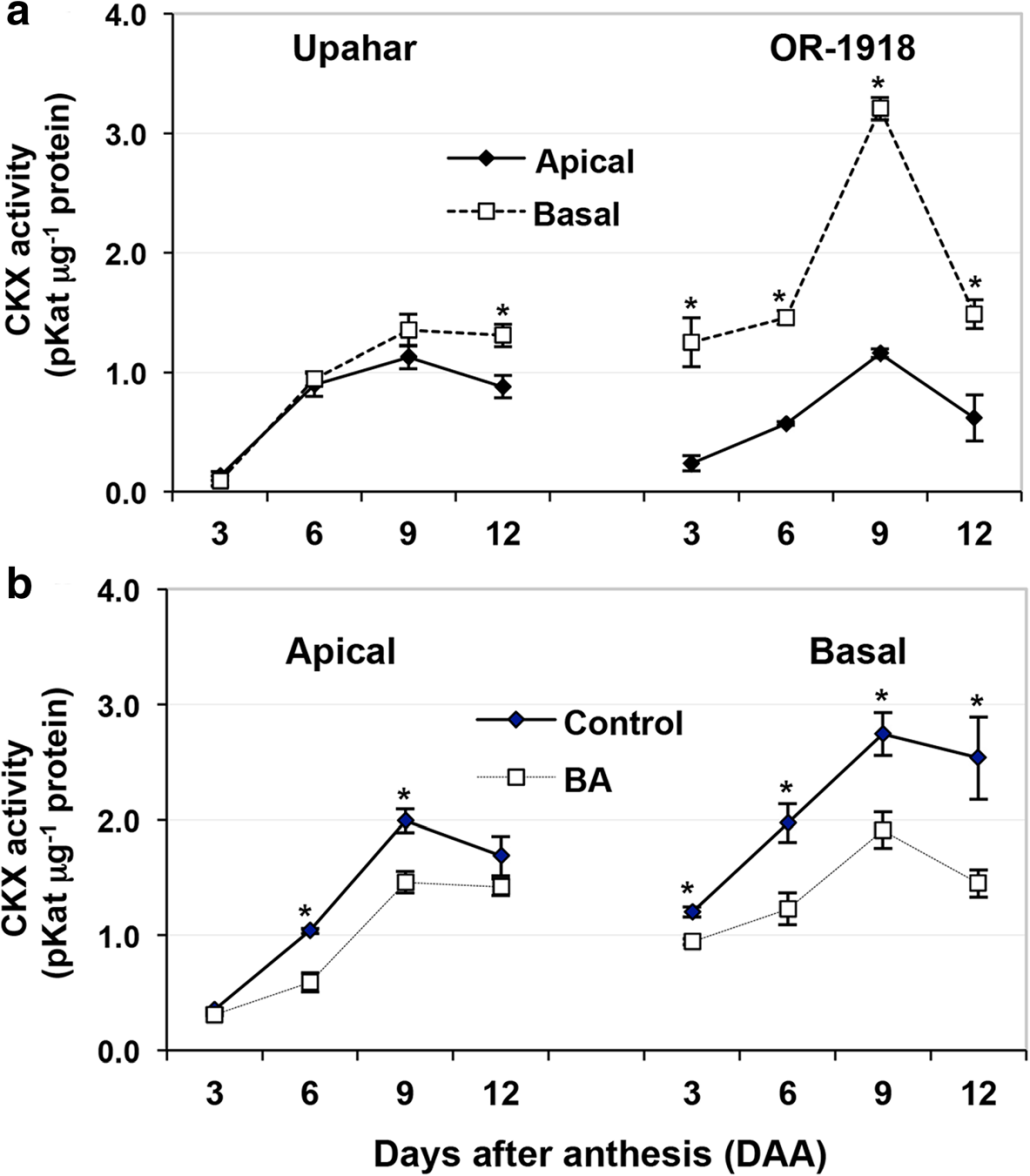
Cytokinin oxidase (CKX) activity in the spikelets sampled on various days after anthesis (DAA): **a**) lax-panicle (Upahar) and compact-panicle (OR-1918) rice cultivars were grown in field and comparative analysis of spatio-temporal changes in the enzyme activity was done between the apical and basal spikelets of the individual cultivars; **b**) the compact-panicle rice cultivar OR-1918 was grown in RCC pots, treated with 50 μM BAP (6-Benzylaminopurine) through root application before heading and comparative analysis of temporal changes in the enzyme activity was done between apical/basal spikelets from the BAP treated and control (not treated with BAP) plants. Each data point is the mean of three independent estimations ± SD. The two data points of the same sampling day marked with ‘*’ differ significantly at least at *p* ≤ 0.05, as seen by ‘t’ test

Great difference in CKX activity was observed between the two cultivars, the lax-panicle Upahar and compact-panicle OR-1918 (Fig. 2a). The activity of CKX was comparatively less in the apical spikelets than that in the basal spikelets in both compact- and lax-panicle cultivars (Fig. 2a). However, the activity of the enzyme differed highly significantly between the apical and basal spikelets of OR-1918 on all the sampling days, which was not observed in Upahar, except on 12 DAA. The activity of the enzyme was especially much high in the basal spikelets compared with that in the apical spikelets in OR-1918 on 9 DAA compared with that on the other days post-anthesis. This was in contrast to that in Upahar in which the activity of the enzyme increased from 3 DAA to 9 DAA gradually before decreasing slightly on 12 DAA in both apical and basal spikelets. It was also observed that the activity of the enzyme in both apical and basal spikelets of Upahar was much less than that in the basal spikelets of OR-1918 on all the days post-anthesis. Exogenous cytokinin (BAP) application significantly decreased the activity of the enzyme in both apical and basal spikelets of OR-1918 (Fig. 2b). The decrease was, however, more drastic in the basal compared with that in the apical spikelets, especially on 9 and 12 DAAs.

**Fig. 3:**
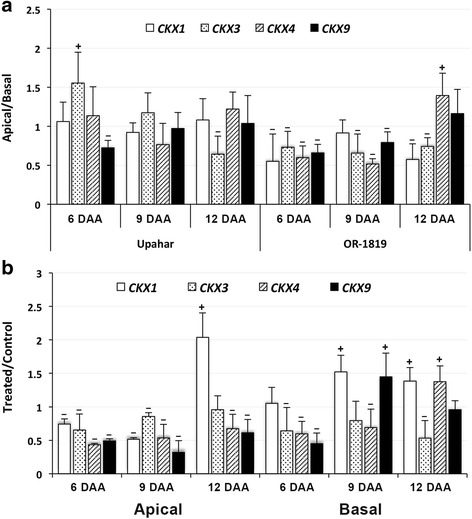
RT-qPCR results representing relative levels of expression of cytokinin oxidase (*CKX*) isoforms in the spikelets sampled on various days after anthesis (DAA) from rice cultivars grown in field (**a**) and in pots (**b**): **a**) relative expression in apical spikelets over basal spikelets; **b**) relative expression in the apical and basal spikelets of the BAP treated plants over that of the control plants. *Actin* served as the reference control. Other experimental details as in Fig. 2. Plus (+)/minus (−) signs against the vertical bars representing individual genes indicate significantly greater/lesser relative expression of the gene in the apical spikelets compared with the basal ones (**a**), or in spikelelets sampled from the treated plants compared with that sampled from the control plants (**b**) at least at *p* ≤ 0.05, as determined by ‘t’ test

No significant difference in the expression of *CKX* was observed between the apical and basal spikelets in Upahar for most of the isoforms studied (Fig. 3a). Only *CKX9* and *CKX3* showed significant difference in expression between the apical and basal spikelets; compared with basal spikelets the expression of *CKX3* was significantly higher on 6 DAA and significantly lower on 12 DAA, and that of *CKX9* was significantly lower on 6 DAA in the apical spikelets. In contrast, the expression of all the *CKX* isoforms on most of the days of sampling was significantly lower in the apical than in the basal spikelets in OR-1918, except of *CKX4* on 12 DAA when the expression of this isoform was significantly more in the apical than in the basal spikelets. All the isoforms of *CKX* showed significant decrease in their expression upon cytokinin treatment in the apical spikelets of OR-1918, except *CKX1* on 12 DAA when the expression of this isoform increased significantly in response to the cytokinin application (Fig. 3b). In contrast, the basal spikelets showed variable response to the cytokinin application. The expression of *CKX3*, *CKX4* and *CKX9* decreased significantly upon cytokinin application on 6 DAA. On 9 DAA sampling the expression of *CKX1* and *CKX9* was significantly higher in the treated case compared to the control, whereas the expression of *CKX3* and *CKX4* decreased in response to cytokinin treatment, and the decrease was significant for *CKX4*. On 12 DAA on the other hand the expression of *CKX4* increased significantly together with significant increase in expression of *CKX1* and decrease in expression of *CKX3* in response to cytokinin application. The expression of *CKX9* also increased upon cytokinin application on 12 DAA, similar that on 9 DAA, but the increase was not significant.

**Fig. 4:**
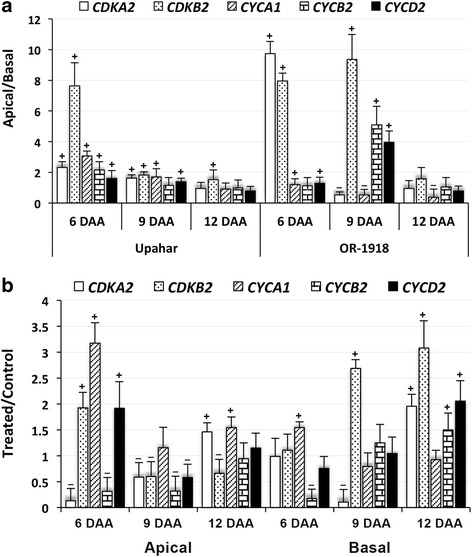
RT-qPCR results representing relative levels of expression of various cell cycle regulator genes in the spikelets sampled on various days after anthesis (DAA) from rice cultivars grown in field (**a**) and in pots (**b**). Other details as in Fig. 3

All CDKs and cyclins (CYCs) exhibited significantly higher expression in the apical than in the basal spikelets on 6 and 9 DAA in Upahar with *CDKB2* showing conspicuously high expression in the apical spikelets on 6 DAA (Fig. 4a). On 12 DAA, the difference in expression of most of them in the apical and basal spikelets was insignificant, except of *CDKB2*, which showed significantly greater expression in the apical compared with the basal spikelets. In contrast to Upahar, OR-1918 showed very high expression of both *CDKs* on 6 DAA and of *CDKB2*, *CYCB2* and *CYCD2* on 9 DAA in the apical than in the basal spikelets. The expression of all cyclins on 6 DAA, *CDKA2* and *CYCA1* on 9 DAA, and all *CDKs* and *CYCs* on 12 DAA differed between the apical and basal spikelets either insignificantly or significantly but with low margin.

Cytokinin treatment significantly increased the expression of *CDKB2*, *CYCA1* and *CYCD2* highly in the apical spikelets on 6 DAA, while significantly decreased the expression of *CDKA2* and *CYCB2* (Fig. 4b). The expression of all these, except of *CYCA1*, on the other hand significantly decreased in the apical spikelets compared to control on 9 DAA. On 12 DAA, however, the expression of all except of *CDKB2* increased again in the apical spikelets in response to the cytokinin application. The basal spikelets showed a trend of increase in expression of *CDKs* and *CYCs* from 6 to 12 DAA upon cytokinin treatment in contrast to the apical spikelets. The expression of only *CYCB2* on 6 DAA and of only *CDKA2* on 9 DAA showed significant decrease in response to cytokinin treatment in the basal spikelets. The expressions of CYCA1 on 6 DAA, of *CDKB2* on 9 DAA and of *CDKA2*, *CDKB2, CYCB2* and *CYCD2* on 12 DAA on the other hand increased significantly in response to cytokinin treatment in the basal spikelets.

**Fig. 5:**
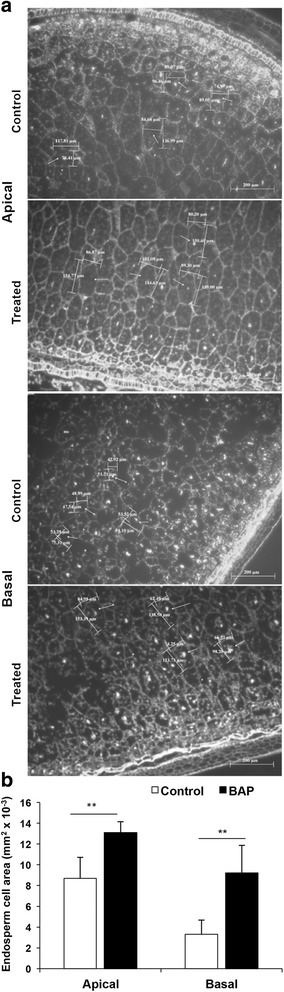
Visualization of endosperm cell size and cell area. Paraffin embedded caryopses from 12 DAA spikelets were sectioned longitudinally by microtome followed by DAPI staining for comparative visualization of cells and nuclear size (**a**), and quantification of the endosperm cell area (**b**) in OR-1918 rice cultivar grown in pots and treated with 50 μM BAP through root application. Four cells from each slide were taken for calculation of cells area and represented as mean (vertical bars) ± SD (**b**). The bar pair marked by ‘**’ indicates significant difference (*p* ≤ 0.01) in the endosperm cell size of the caryopses from control and BAP treated plants, as revealed by ‘t’ test (**b**)

**Fig. 6:**
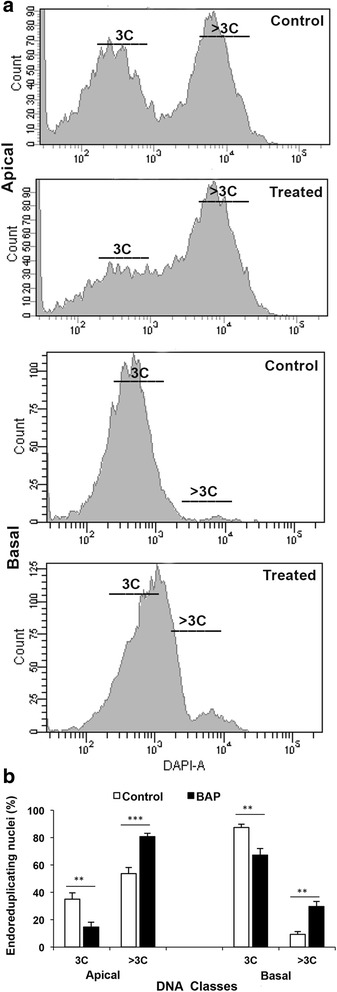
Flowcytometric analysis of ploidy (DNA class, 3C or > 3C) status of the endosperm nuclei in the apical and basal spikelets sampled on 12 DAA from control and 50 μM BAP treated rice cultivar OR-1918 (**a**), and quantification of the ploidy status of nuclei (**b**). The endosperm cells of the caryopses released in solution by cellulase treatment were stained with 4′,6-diamidino-2-phenylindole (DAPI) for counting the cells (nuclei) representing various ploidy levels (3C and > 3C). Each panel is representative of three counts from independent endosperm cell preparations. The vertical bars represent mean ± SD of ploidy status of the nuclei in three independent endosperm cell preparations. The bar pair marked by ‘**’ and ‘***’ indicate that the percentage of nuclei representing a ploidy status (3C or > 3C) in the endosperm cells from control and BAP treated plants differed significantly at *p* ≤ 0.01 and *p* ≤ 0.001, respectively, as revealed by ‘t’ test (**b**)

Cytokinin treatment increased the endosperm cell size in both apical and basal spikelets (Fig. 5a, b). The increase in the endosperm cell area was statistically significant in both the cases (Fig. 5b). However, the increase in the endosperm cell size in response to cytokinin treatment was more in the endosperm of the basal than in the endosperm of the apical spikelets compared to the respective control (Fig. 5b). The increase in the cell size in response to the cytokinin application was also accompanied by increase in ploidy level of the nuclei, both in the apical and basal spikelets, as is evident from the flowcytometric data (Fig. 6a). In the apical spikelets the nuclei with ploidy level 3C and > 3C were present in nearly equal numbers (Fig. 6a), but the cytokinin treatment created a great shift in their ploidy level to >3C (Fig. 6b). In the basal spikelets, the percentage of nuclei with 3C was much more than that with >3C ploidy, but the difference was significantly reduced upon cytokinin treatment that promoted endoreduplication (Fig. 6b).

**Fig. 7:**
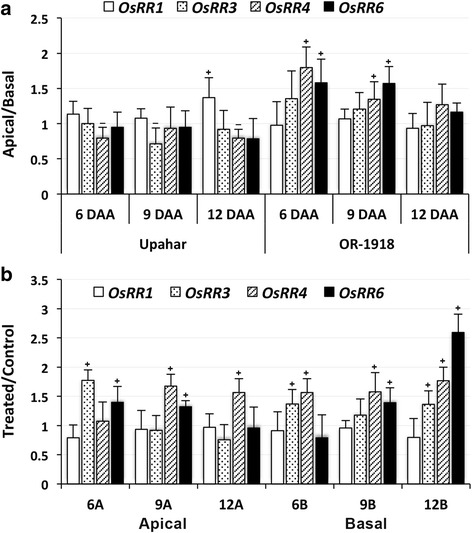
RT-qPCR results showing relative levels of expression of various response regulator genes (*OsRRs*) in the spikelets sampled on various days after anthesis (DAA) from rice cultivars grown in field (**a**) and in pots (**b**). Other details as in Fig. 3

Among the cytokinin signaling components, the expressions of four response regulators, namely *OsRR1*, *OsRR3*, *OsRR4* and *OsRR6*, were recorded (Fig. 7a). They did not show any trend in spatio-temporal variation in either of the cultivars. The expressions of most *OsRRs* in Upahar showed insignificant difference whether between apical and basal spikelets or between different days after anthesis except that the expression of *OsRR1* was significantly higher in the apical spikelets compared to that in the basal spikelet on 12 DAA, and the reverse was the case for *OsRR4* on 6 and 12 DAA and for *OsRR3* on 9 DAA. In contrast to Upahar, the expression of *OsRR4* and *OsRR6* in OR-1918 was significantly greater in the apical over the basal spikelets on 6 and 9 DAA, and the difference became insignificant on 12 DAA. The difference in expression of other *OsRRs* between the apical and basal spikelets remained insignificant on these days. Cytokinin treatment had an accelerating effect on the expression of most *OsRRs* (Fig. 7b). It significantly accelerated the expression of *OsRR3* on 6 DAA, of *OsRR4* on 9 and 12 DAA and of *OsRR6* on 6 and 9 DAA in the apical spikelets. Cytokinin application had a greater accelerating effect on the expression of *OsRRs* in the basal spikelets in comparison to that in the apical spikelets; it significantly accelerated the expression of *OsRR3* on 6 DAA and 12 DAA, of *OsRR6* on 9 and 12 DAA and of *OsRR4* on all DAAs in the basal spikelets.

**Fig. 8:**
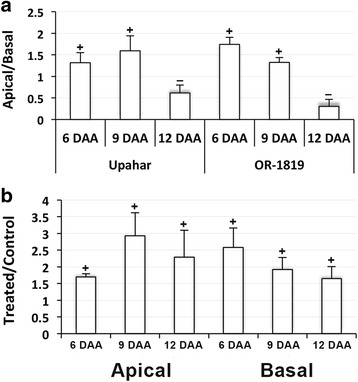
RT-qPCR results showing relative levels of expression of sucrose synthase3 (*SUS3*) in the spikelets sampled on various days after anthesis (DAA) from rice cultivars grown in field (**a**) and in pots (**b**). Other details as in Fig. 3

In order to understand the reason of enhancement in grain filling in response to the application of cytokinin, expression study of the main isoform of *SUS* (sucrose synthase), *SUS3,* was conducted in the spikelets. *SUS3* expressed significantly more in the apical than in the basal spikelets on 6 and 9 DAA, but significantly less in the apical compared with that in the basal spikelets on 12 DAA in both Upahar and OR-1918 (Fig. 8a). Cytokinin treatment increased the expression of *SUS3* significantly in the apical as well as in the basal spikelets on all days of sampling (Fig. 8b). However, the influence of the treatment was more on 9 and 12 DAA in apical spikelets and on 6 DAA in the basal spikelets.

**Fig. 9:**
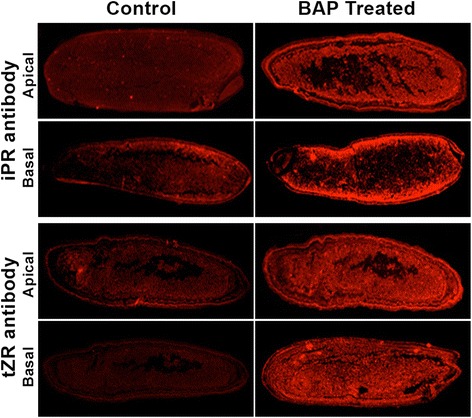
Immunodetection of trans-zeatin (tZ) riboside (tZR) and N^6^-(Δ^2^-isopentenyl)adenine (iP) riboside (iPR) in the endosperm cells of the spikelets sampled on 9 DAA from control and 50 μM BAP treated rice cultivar OR-1918. Immunolabeling for isopentenyladenosine (iPR) and tans-zeatin riboside (tZR) was done by polyclonal antibodies raised against iPR/tZR conjugated with BSA, and immunodection followed incubation with secondary antibody. The longitudinal sections of the whole caryopses were observed under a fluorscence streo-microscope

**Fig. 10:**
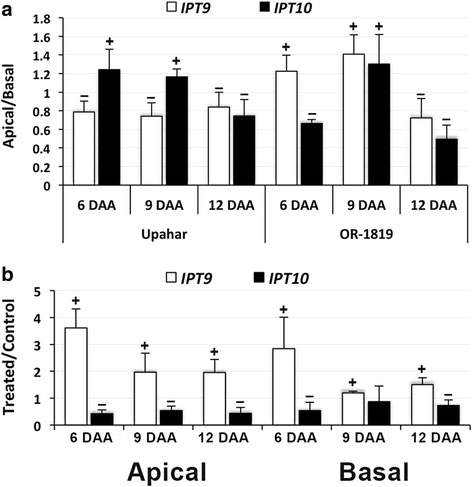
RT-qPCR results showing relative levels of expression of two isopentenyl transferases (*IPTs*) in the spikelets sampled on various days after anthesis (DAA) from rice cultivars grown in field (**a**) and in pots (**b**). Other details as in Fig. 3

Exogenous application of cytokinin to OR-1918 also influenced the endosperm cellular level of the hormone. This is reflected from the increase in the level of N^6^- isopentenyladenosine (iPR) and trans-zeatin riboside (tZR), and possibly also their bases (iP and tZ), as revealed by histochemical analysis of the sections of the caryopses probed with iPR and tZR antibodies, respectively (Fig. 9). The increase in their level was visible in the caryopses of both the apical and the basal spikelets, but the increase was comparatively more in the caryopses from the basal spikelets compared with that from the apical spikelets. In order to understand the reason of their increase, expression studies of isopentenyl transferases (*IPTs*) were carried out. Out of the two isoforms of *IPT*, including *IPT9* and *IPT10*, that were found expressing in the two rice cultivars, *IPT9* expressed significantly less in the apical spikelets compared with that in the basal spikelets in Upahar on all the days of sampling, whereas the result was reverse for *IPT10*, except on 12 DAA (Fig. 10a). In contrast to Upahar, OR-1918 showed significantly greater expression of *IPT9* in the apical spikelets compared with the basal ones on 6 and 9 DAA and significantly lower expression of *IPT10* in the apical spikelets than that in the basal spikelets on 6 DAA. The expressions of both *IPT9* and *IPT10* were significantly lower in the apical than in the basal spikelets in OR-1918 on 12 DAA, similar to that in Upahar. Cytokinin application increased the expression of *IPT9* significantly in both apical and basal spikelets on all the days of sampling (Fig. 10b). In oppose to *IPT9*, the expression of *IPT10* decreased significantly in both apical and basal spikelets on all the sampling days except on 9 DAA.

## Discussion

It is well established that cytokinins (CKs) have stimulatory effect on cell division in both vegetative and reproductive tissues of plants, as is reflected from their high level in mitotically active areas, like that in the root and shoot meristem [52] and endosperm [44, 45]. So far as reproductive tissue is concerned, there are a few reports of increase in grain weight and endosperm cell numbers in rice upon kinetin application through root treatment [35, 41, 43, 44]. The increase in grain filling in the basal spikelets of OR-1918 observed in this study (Table 1) could be a result of stimulatory effect of cytokinin (CK) on the endosperm cell number, which has been reported to bear positive correlation with grain filling and grain weight [53]. CK has also been reported to bear positive relation with the number of spikelets on a panicle in rice [19], as well as in wheat [54], similar to that found in this study (Table 1). Taken together, CK can be considered as an important agent in enhancing the yield potential of rice, as is reflected from its effect on yield value per plant that increased significantly (Table 1).

The function of CK in cell proliferation, cell division and differentiation, and shoot and root growth has been well documented [55–57]. The first line of evidence in this direction came from overexpression of *CKX*, which degrades active CKs, in *Nicotiana tabacum* that resulted in cytokinin-deficient plants with smaller apical meristems and stunted shoots [28]. The second line of evidence came from identification of QTL for grain number in rice panicle that encode *CKX* (*OsCKX2*), the expression of which bears negative correlation with grain number on panicle indicating a positive or stimulatory role of CK on grain number [19]. The increase in the number of grains per panicle upon CK treatment in OR-1918 (Table 1) also supports its role in increasing grain number in rice panicle. However, report on possible influence of CK on grain filling is scant, although it is well established that CKs level is abundant in immature seeds [58], and prior to grain filling the endosperm cell enters into syncytium phase followed by cellularization [59] and increase in cell numbers [53]. Significantly higher activity level of CKX in the basal spikelets compared with that in the apical spikelets of the compact-panicle rice cultivar OR-1918 (Fig. 2A) coupled with differential filling of grains in them (Table 1) also indicates that the poor filling of grain in the basal than in the apical spikelets might be related to the level of the hormone in their endosperm cells. A study of CK levels in maize caryopses has in fact revealed highest peak between 8 and 12 DAP (days after pollination), the active period of grain filling, followed by a drop of CK content at 16 DAP [60]. A sharp increase in endogenous CKs has been reported during the phase of active division of endosperm cells in the developing grain in wheat [61]. In general it has been seen that enhancing CK biosynthesis increases yield attributes like pod/seed set and seed development [57]. Besides, it has also been seen that poor grain filling in the basal spikelets in response to drought stress to the plants is associated with a low level of CKs in their endosperm cells when compared to that in the endosperm cells of the apical spikelets in which the grain filling is not affected significantly by the stress [62]. A very high activity of CKX on 9 DAA in the basal spikelets of OR-1918 thus gives an indication that poor filling of grain in them could be as a result of low CK level in comparison to that in the apical spikelets. The present inverse relationship between grain filling and CKX activity is also supported by the observation of an inverse correlation between 1000 grain weight and *CKX* expression in seven varieties of wheat [63].

The homeostasis of CKs in cells and tissues is maintained not only by its biosynthesis by the enzymes like IPTs (adenosine phosphate isopentenyl transferases) and CYP735A, but also by their degradation by CKX [57] and conversion to inactive *O*-glucosides and *N*-glucosides [31, 64]. The expressions of CKs biosynthesis enzymes are in turn regulated by the phytohormones like ABA and auxin, and CK itself [56]. Exogenous CK (N^6^-benzylaminopurine, BAP) treatment has been reported to accelerate CKX activity in a few studies involving plant organ and plant culture [65, 66]. The reason of such cytokinin-induced increase in CKX activity has been suggested to be the need of re-establishment and maintenance of intracellular CK homeostasis [67] with the enzyme playing a role as a “detoxifier” of cytokinins in case availability of the hormone is in excess [68]. The exogenous application of CK in the present study, however, resulted in decrease in CKX activity both in apical and basal spikelets (Fig. 2B), suggesting that response to exogenous cytokinin application may be different at the level of tissue (in vitro) than in whole plant (in vivo). The decrease in CKX activity level upon exogenous application of CK observed in the present study supports the maintenance of a high level of the compound in the spikelets of rice plants exposed to the hormone, as reported by others [35, 44], although the study does not agree with the observation of increase in the activity of CKX in response to CK at the level of tissue/plant culture [65, 66]. The reason of difference in response of CKX in vegetative and reproductive tissues to exogenous application of cytokinin to plants could be because the two tissues might be following different mechanisms for regulation of CK homeostasis in them. Several studies have shown that CKs are synthesized in roots and carried through xylem to the aerial parts, including leaves and reproductive organs with the transpiration system [69–72], and senescence and abscission of leaves is initiated with decrease in synthesis and supply of the hormone from root, the signal for which is sent from shoot to root in some form, the chemical nature of which is so far not clear, when the seeds are in the late stage of development [70, 71, 73–75]. However, synthesis of the hormone de novo in leaves has also been indicated for transportation through phloem to the shoot apex and major sinks lacking xylem connectivity [72, 76]. Because of similar restriction of transport of CKs to the floral organs, maintenance of a low activity of CKX and its proper regulation might be important for maintaining the required level of the hormone in them. This is in conformity with the proposed role of CKX in maintenance of an optimal level of the hormone for growth and/or maintaining the cytokinin signaling system to a required level [28].

Rice genome has 11 isoforms of *CKX* gene, *OsCKX1*-*OsCKX11*, of which *CKX2*, *CKX3*, *CKX4*, *CKX5*, *CKX9* and *CKX11* were found to be prominently expressing in rice inflorescence [19]. However, the current study found prominent expression of only four isoforms, including *CKX1*, *CKX3*, *CKX4* and *CKX9* in the rice spikelets (Fig. 3a). Interestingly, the isoform *CKX2*, which expressed in flowers and inflorescence meristem of *O. sativa* Japonica cv. Koshihikari bearing less number of spikelets but not in *O. sativa* Japonica cv. 5150 bearing large number of spikelets, indicating a possible inverse relationship of the enzyme with number of spikelets on a panicle by regulation of the cellular cytokinin level [19], was not found to be expressing either in Upahar bearing less number of spikelets or in OR-1918 bearing large number of spikelets. This suggested that the QTL for grain number, Gn1a, harbouring the candidate gene *CKX2* may not be the only factor or gene determining grain numbers in Indica rice cultivars. However, present study does suggest that *CKX* isoforms like *CKX1*, *CKX3*, *CKX4* and *CKX9* could be playing important role in Indica cultivars with regard to grain filling. This is because these *CKX* isoforms had more or less similar expressions in the apical and basal spikelets of the lax-panicle cultivar Upahar (Fig. 3a) together with virtually no difference in the activity of CKX in them (Fig. 2a). Furthermore, these *CKX* isoforms had significantly lower expressions in the apical than in the basal spikelets of the compact-panicle cultivar OR-1918 on most DAAs (Fig. 3a), which was reflected as significantly low activity of CKX in the apical than in the basal spikelets (Fig. 2a).

The observed downregulation of expression of the *CKX* isoforms in OR1918 in both apical and basal spikelets upon CK application (Fig. 3b), at least on 6 DAA, the beginning of active phase of endosperm cell division [53], although emphasizes their important role in regulation of the endosperm CK level leading to improvement in grain filling, it is in oppose to the reports of increase in activity of CKX in vegetative tissues in response to exogenous application of the hormone [65, 66]. Besides, using isolated developing kernels and leaf disc of *Zea mays* Brugiere et al. [68] have also shown *CKX* to be inducible by exogenous cytokinins. These observations further suggest that the CK homeostasis mechanism in the reproductive organ in vivo could be different from that operational in the vegetative organs or explants, or in the organs in which the transport of the hormone from root is smooth. Taylor et al. [77] indicated that it is the CKs present in the phloem sap coming from the leaves that enter into the developing inflorescence and fruits rather than that present in xylem coming from roots. In addition, calculation by Letham et al. [78] has shown that only 1.1% of CKs in seed are derived directly from the xylem. However, it is not yet established what leads to the build up of the required level of CKs for the overall growth of the caryopses to mature seed, although temporal variation of the hormone during endosperm development and seed maturation is well established [35, 44, 62, 79, 80]. Upon exogenous application of cytokinin an increase in its level in the endosperm cells may be anticipated through phloem transport, although the same has not been reported so far. An increase in the levels of the hormone in the endosperm cells in fact may also be anticipated based on the available reports that exogenous cytokinin application to rice seedlings induces expression of most of *OsRRs* [46], and that overexpression of *OsRR6* in rice leads to downregulation of *OsCKX4* and *OsCKX5* [75]. However, the mechanism of maintenance of cellular CK homeostasis mediated through type-A RRs regulated expression of *CKX* is yet to be fully understood, particularly in developing endosperm cells. This is because there are 13 *OsRRs* in rice and the regulatory role of each of them is yet to be characterized, although it is established through transgenic experiments that type-A RRs act as negative regulator of CK signaling [81], and overexpression of type-A RRs, like *ARR4*, *ARR5*, *ARR6* and *ARR7* represses the promoter activity of *ARR6* [82]. Significant acceleration in expression of *CKX1* and *CKX9* on 9 DAA and of *CKX1* and *CKX4* on 12 DAA in response to the exogenous CK application might be because of above transcription regulatory mechanism of the *CKX* genes not yet understood fully. Irrespective of the factor(s) regulating the expression of individual *CKX* isoforms, it is, however, clearly established that a low CKX activity favours grain yield, both in terms of grain number and grain weight, as silencing of *HvCKX1* in barley increased grain yield of the plant [29], similar to that obtained with mutation of *CKX2* in rice [19]. Our findings in the present study (Fig. 2a, b) are in conformity to these reports [19, 29].

It is well established that grain filling process is preceded by endoreduplication of endosperm cells regulated by cell cycle regulators [83], and CK plays important role in cell division, proliferation and differentiation [55–57]. A few reports have demonstrated that CK induces expression of *CYCD3* [84, 85], and that activation of CDKA by type-D cyclins (CYCDs) promotes G1-S transition in plants leading to active cell division [86]. Type-B cyclins on the other are the cyclin class that is involved in G2-M phase transition through interaction with CDKA and CDKB [52, 86]. Although G2-M phase transition is regulated in plant by CK application, as evidenced by several experiments on cell lines [87, 88], the mechanism is not yet fully clear, except that cytokinin can be replaced by the transgene *CDK-Tyr* phosphatase Cdc25 for supporting and initiating mitosis [89]. The possible role of cyclins and CDKs in cell division is clearly visible in the present study also, as OR-1918 had a very high level of expression of *CYCA1*, *CYCB2* and *CDKB2* in the apical spikelets compared with that in the basal spikelets on 9 DAA (Fig. 4A) representing initiation of highly active phase of endosperm cell division and corroborating with the fact that the apical spikelets had better grain filling than the basal spikelets (Table 1). On the other hand in Upahar that shows no difference in grain filling between apical and basal spikelets (Table 1), the spatial difference in the expression of these cell cycle regulators on 9 DAA was not much pronounced, although significant (Fig. 4a). Hence, it is possible that highly significant accelerating effect of CK treatment on the expression of *CDKs* and *CYCs* in apical and basal spikelets, particularly on 12 DAA in the basal spikelets (Fig. 4b), might be causing an enhanced division of endosperm cell in them during the period leading to significant increase in grain filling (Table 1).

Grain filling is although positively related to endosperm cell division [53, 54], it is also a result of increased anabolism symbolized by increase in cell size and supported by endoreduplication of the nuclei, a phenomenon widely reported for storage tissues and other tissues undergoing rapid differentiation and expansion in plants like that in root nodules, cotyledons and pericarp [90–93]. The increase in grain filling in OR-1918 upon CK application in the present study also appears to be a result of increase in cell size and endoreduplication (Fig. 5a, b, 6a, b). It has been suggested that endoreduplication, which triggers anabolism that follows increase in cell size, is a result of check of G2-M phase transition. This blockage results in re-entry of the cells to S phase leading to increase in their ploidy level. The check of G2-M transition is although suggested to be primarily a result of decrease in cyclin B level [83], it is not supported in this study since the expression of cyclin B significantly increased in both apical and basal spikelets upon cytokinin treatment, particularly on 12 DAA (Fig. 4b), the approximate start time point of endoreduplication process. The process of endoreduplication thus could be more complicated than simply to be a result of depletion of the cyclin B level in the cells, particularly in context to the role of CK, or plant hormone in general. This is reflected from the findings like 1) CKs inhibit endocytic recycling of PIN1, a member of PIN family of auxin efflux carriers that promotes degradation of auxin in the vacuole, leading to increase in its cellular level and thereby promoting endoreduplication [88, 94, 95], 2) CKs promotes expression of *CCS52A1* that stimulate degradation of mitotic cyclins, which promotes endoreduplication [96], and 3) endogenous oscillation of CKs level accompanies progress of cell cycle, but their exogenous application that decreases the amplitude of fluctuation of the hormone retards cell cycle progression, promoting endoreduplication [97]. In general it is said that CKs at lower concentrations activates mitosis and cell division, but at higher concentrations favour endoreduplication [86].

Cytokinin response in rice is mediated by two-component systems involving histidine kinase membrane bound receptors, OHKs, and multistep phosphorelay signaling components comprising of histidine-contaning phosphotransmitter (OHP) and response regulators (RR), which are categorized into type-A, OsRR, and type-B, ORR [27]. CKs are perceived by histidine kinases generating signal that is transferred in the form of phosphoryl group through OHP in the cytoplasm to the response regulators in nucleus [98, 99]. Five histidine kinases, *OHK1*-*OHK5*, two histidine phosophotransmitter, *OHP1* an *OHP2*, thirteen type-A *RR*, *OsRR1*-*OsRR13*, and five type-B *RR*, *ORR1*-*ORR5* genes have been identified in rice through database search of Japonica rice using the sequence information of such genes from *Arabidopsis* and maize [27]. Expressions of all these genes, except of *OsRR1–3* and *OsRR5–7*, were checked in different tissues of rice, and were found to be expressing [27]. However, the current study was restricted to see the expression pattern of only *OsRRs* of the two component signaling system. This is because genes that code for HK, HP and type-B RR have been reported not to be responsive to CK [75]. Besides, it has also been seen that CK regulates the expression of type-A *RR* in addition to a large number of other genes [75, 100, 101]. In fact, type-A *RRs* have been reported to be the primary CK response genes since these are induced by the hormone even in the absence of protein synthesis de novo [46]. Besides, overexpression of a type-A RR member, *OsRR6*, in rice abolishes shoot regeneration, suggesting a negative regulator function of *OsRR* in CK signaling [75]. The role of type-A *RR* as negative regulator in CK signaling has also been demonstrated in *Arabidopsis* by overexpression of *ARR8* and *ARR15* in the plant [82, 102]. However, specific role of the individual *OsRRs* is yet to be studied. Besides, there occurs cultivar-specific differences in expression of the *OsRRs*; while Jain et al. [46] reported expression of all the *RR* isoforms in flower of the Indica cultivar Pusa Basmati, Ito and Kurata [27] did not find *OsRR1–3* and *OsRR5–7* to be expressing in Japonica cultivar Nipponbare. The current study is in agreement to this, as only *OsRR1*, *OsRR3*, *OsRR4* and *OsRR6* were found to be mainly expressing in the spikelets of OR-1918 and Upahar (Fig. 7a). The other isoforms although expressed, but their expression was either very low or these expressed only on the initial days of anthesis (data not shown).

The comparative expression study of the four *OsRR* isoforms in apical and basal spikelets of Upahar and OR-1918 (Fig. 7a) suggested that a high expression level of *RRs* might favour grain filling. This is because the expressions of at least *OsRR4* and *OsRR6* in OR-1918 showing spatial difference in grain filling were more in the apical than in the basal spikelets (Fig. 7a), which might be a result of a greater phosphorylation status of the pool of type-B RR in the presence of a higher level of CKs in the apical than in the basal spikelets (Fig. 9); it is well established that upon getting phosphorylated by histidine-containing phosphotransmitter, type-B RRs activate transcription of the primary cytokinin response genes, including some type-A RRs [82, 101, 103]. Comparatively poor expression of *OsRR4* and *OsRR6* in the basal spikelets than in the apical ones could be the reason of a higher activity and expression of *CKX* in the basal spikelets compared with that in the apical spikelets of OR-1918 (Figs. 2a, 3a) leading to differential grain filling in them; transcriptional repressor function of certain type-A RRs has been reported [82]. Overexpression of at least two type-A RRs, *ARR7* and *OsRR6*, have been found to lead to downregulation of *CKX* in *Arabidopsis* and rice, respectively [75, 104]. In addition, it is well established that type-A RRs are negative regulators of CK responses [81, 82]. However, the factors that determines differential expression of type-A RR in tissues of the same plant, the apical and basal spikelets in the present case, in absence of exogenous application of CK is not yet established. The tissue/organ based differential expression of type-A *RRs* is reflected in the present study even in response to exogenous application of CK with the basal spikelets showing greater response in terms of induced expression of *OsRRs* than the apical spikelets (Fig. 7b). Besides, the response of the individual *RRs*, including *OsRR3*, *OsRR4* and *OsRR6*, varied depending on the days after anthesis with *OsRR1* not showing any response (Fig. 7b). Although the reason of spatio-temporal differential response of these *RRs* to CK application may not be explained, the results do indicate that increase in the expression level of *OsRR3*, *OsRR4* and *OsRR6* might be the cause of improvement in grain filling in OR-1918, particularly in the basal spikelets, brought about by decrease in expression of *CKX* (Fig. 3b). Improvement in grain filling induced by cytokinin application is also reflected from upregulation of *SUS3* expression, the major isoform of sucrose synthase that drives the initial reaction leading to conversion of sucrose to starch, in the basal spikelets of OR-1918 (Fig. 8b). However, so far no relationship of the cellular CK level or cytokinin signaling with the expression of the enzymes involved in grain filling has been indicated. It could be that cytokinin-induced increase in cell size (Fig. 5a, b) and endoreduplication in the endosperm cells of both apical and basal spikelets of OR-1918 (Fig. 6a, b) might be driving better expression of *SUS3*, and also of other enzymes involved in grain filling.

It is established that the metabolic control of CKs is highly complex, as is reflected from the numbers of genes whose expression is influenced by them [75, 100, 105, 106], and this is further complicated by their structural diversities and the biosynthesis and degradation mechanisms involved in maintenance of their cellular homeostasis [56, 75]. Besides, it is not yet clear what determines the structural variants of CK to be present in cell and in what proportion, which are also species-specific [56]. Exogenous application of synthetic hormone BAP results in great variation in cellular level of various CKs in tobacco and wheat [54, 66], the reason of which is not yet explained. Increase in the levels of isopentenyladenosine (iPR) and trans-zeatin riboside (tZR) in the caryopses in response to exogenous BAP application (Fig. 9) is in agreement with the reports available. The increase in the level of iPR and tZR in response to exogenous application is also supported from acceleration in expression of *IPT9* in both apical and basal spikelets, comparatively more in the apical than in the basal spikelets (Fig. 10b), corresponding to increase in the level of iPR and tZR (Fig. 9). In contrast, the expression of *IPT10*, the other isoform of the enzyme that expressed under control condition, declined in response to BAP application (Fig. 10b), suggesting CK inducibility of *IPT9*. Besides, a greater expression of *IPT9* in the apical than in the basal spikelets of the compact-panicle cultivar OR-1918 (Fig. 10a), coupled with increase in its expression in response to BAP application in both apical and basal spikelets (Fig. 10b) and improvement in grain filling (Table 1) suggested it to be an important factor in determining grain filling in rice.

## Conclusions

The study thus indicated that CK metabolism could be of much importance in the process concerned with grain filling in rice. It is well reflected from a high activity level of CKX and a greater expression of the *CKX* isoforms in the poorly filled basal spikelets compared with that in the well filled apical spikelets of the compact-panicle rice cultivar OR-1918. Furthermore, exogenous application of CK improves grain filling in the spikelets significantly, and this is accompanied by significant decrease in the levels of expression of most of the *CKX* isoforms, both in apical and basal spikelets. The findings not only support the reported negative relationship of CKX activity with grain number [19], but also explain observation of high CK level in immature seeds [58, 60], as both the processes precede an enhanced cellularization, which CK supports [55, 56]. At the level of maintaining cellular homeostasis of cytokinin, it may be said that the hormone acts as repressor of *CKX* expression through type-A RRs, as evidenced by transgenic experiments [81, 82], although the process is far more complex [35, 62, 65, 66, 68, 77]. Moreover, the level of CK in the caryopses might also be modulated through regulation of expression of *IPTs*, particularly of *IPT9*, as its expression level increased significantly in response to BAP application. The study finally suggested that grain filling in rice can be improved through biotechnological intervention, which may be achieved by seeds-specific overexpression of type-A *RRs*, like *OsRR4* and *OsRR6* that showed significantly high expression in the apical than in the basal spikelets, or more specifically by seed-specific overexpression of *IPT9* that showed significant increase in response to exogenous application of CK.

## Additional file

Additional file 1: Nucleotide sequences of the genes considered in this study and the primers used for their expression studies. (XLSX 34 kb)
